# Functional characterization of sodium-pumping rhodopsins with different pumping properties

**DOI:** 10.1371/journal.pone.0179232

**Published:** 2017-07-27

**Authors:** Satoshi P. Tsunoda, Matthias Prigge, Rei Abe-Yoshizumi, Keiichi Inoue, Yuko Kozaki, Toru Ishizuka, Hiromu Yawo, Ofer Yizhar, Hideki Kandori

**Affiliations:** 1 PRESTO, Japan Science and Technology Agency, Kawaguchi, Saitama, Japan; 2 Life Science and Applied Chemistry, Graduate School of Engineering, Nagoya Institute of Technology, Nagoya, Japan; 3 OptoBioTechnology Research Center, Nagoya Institute of Technology, Nagoya, Japan; 4 Department of Neurobiology, Weizmann Institute of Science, Rehovot, Israel; 5 Department of Frontier Materials, Nagoya Institute of Technology, Nagoya, Japan; 6 Department of Developmental Biology and Neurosciences, Tohoku University Graduate School of Life Science, Sendai, Japan; University of Cambridge, UNITED KINGDOM

## Abstract

Sodium pumping rhodopsins (NaRs) are a unique member of the microbial-type I rhodopsin family which actively transport Na^+^ and H^+^ depending on ionic condition. In this study, we surveyed 12 different NaRs from various sources of eubacteria for their electrophysiological as well as spectroscopic properties. In mammalian cells several of these NaRs exhibited a Na^+^ based pump photocurrent and four interesting candidates were chosen for further characterization. Voltage dependent photocurrent amplitudes revealed a membrane potential-sensitive turnover rate, indicating the presence of an electrically-charged intermediate(s) in the photocycle reaction. The NaR from *Salinarimonas rosea* DSM21201 exhibited a red-shifted absorption spectrum, and slower kinetics compared to the first described sodium pump, KR2. Although the ratio of Na^+^ to H^+^ ion transport varied among the NaRs we tested, the NaRs from *Flagellimonas* sp_DIK and *Nonlabens* sp_YIK_SED-11 showed significantly higher Na^+^ selectivity when compared to KR2.

All four further investigated NaRs showed a functional expression in dissociated hippocampal neuron culture and hyperpolarizing activity upon light-stimulation. Additionally, all four NaRs allowed optical inhibition of electrically-evoked neuronal spiking. Although efficiency of silencing was 3–5 times lower than silencing with the enhanced version of the proton pump AR3 from *Halorubrum sodomense*, our data outlines a new approach for hyperpolarization of excitable cells without affecting the intracellular and extracellular proton environment.

## Introduction

The microbial rhodopsins are a class of membrane proteins with seven transmembrane helices harboring mainly an all-*trans* retinal chromophore which is covalently bound to a side chain of a lysine residue via Schiff base. They are widely distributed through eukaryotic and prokaryotic organisms and exhibited diverse, specialized functions. Their molecular activity involves active ion transport (ion pumps such as bacteriorhodopsin), passive ion conductance (ion channels, e.g. channelrhodopsins), a light sensation which initiates signal transduction for phototaxis responses (like sensory rhodopsin I and II), and several enzymatic activities such as cyclase [1–3]

Discovery of the first ion pumping rhodopsin, bacteriorhodopsin (BR), in 1971, attracted significant scientific attention to this class of membrane proteins [4]. The photochemical properties as well as proton pumping mechanism were long studied and now the proton transport pathway is established at an atomic resolution [5–7]. Electrophysiological methods were used to understand the relationship between electrochemical gradients, such as membrane potential (dΨ) and transmembrane proton gradient (dpH), and pumping efficiency. From the current-voltage behavior, proton pumping rate of BR is linearly accelerated over the measurable range between -160 and +60 mV [8,9]. Thus the photocycle of BR involves dΨ dependent steps i.e. proton.

uptake from cytoplasmic side, and transition between M1 and M2. On the other hand, the influence of dpH is rather small in the physiological pH range. However, a study from another H^+^ pumping rhodopsin from *Acetabularia*, which is highly homologoues to BR, reported that the photocurrent is increased at pH 10, implying a dependence of deprotonation step of carboxyl residue(s) as a molecular determinant of photocycle speed [10]. Influence of dpH on H^+^ pumping was also demonstrated for proteorhodopsin and *Gloeobacter* rhodopsin [11,12].

Sodium pumping rhodopsins (NaRs) were identified from many flavobacteria [13–15]. They actively transport Na^+^ in Na^+^-containing solution, and H^+^ in the absence of Na^+^ in the solutions. Spectroscopic studies revealed that the first discovered NaR from *Klokinobacter eikastus* (KR2) is 8000 times more selective for H^+^ than Na^+^. KR2 transports mainly Na^+^ under physiological condition where [Na^+^] is much higher than [H^+^] (i.e. [Na^+^]/[H^+^] >10^6^) [16]. Spectroscopic and X-ray studies revealed the cation transport pathway of KR2 [17,18], demonstrating that upon photoisomerization of all-*trans* retinal, a protonated Schiff-base provides H^+^ to D116 which breaks an electrical barrier. This change allows Na^+^ to pass through the chromophore region of the protein. After re-isomerization, H^+^ moves back to the retinal Schiff base to rebuild the electric barrier, preventing Na^+^ from re-entering the chromophore site. Thus, vectorial transport is achieved. Voltage-clamp measurement in primary neurons further demonstrated that the current-voltage relationship is nearly flat between -70 mV and +50 mV, indicating that the photocycle is independent from applied voltage under these experimental conditions [17].

To gain a more precise understanding of the biophysical properties of NaRs, we perform electrophysiological measurements on KR2 expressing mammalian cells. Based on the functional expression of KR2 under these conditions we set out to identify new NaRs with regards to a high expression in plasma membranes of mammalian cells as well as large photocurrent amplitudes.

## Materials and methods

### Phylogenic analysis of rhodopsin genes

The amino acid sequences of rhodopsins were aligned using MUSCLE program [19] after the removal of weakly conserved interhelical loop, and N- and C-terminal extensions in order to increase the accuracy of alignment. The evolutionary history was inferred using the Neighbor-Joining method [20]. The percentage of replicate trees in which the associated taxa clustered together in the bootstrap test (1000 replicates) were calculated [20]. The tree is drawn to scale, with branch lengths in the same units as those of the evolutionary distances used to infer the phylogenetic tree. The evolutionary distances were computed using the Poisson correction method and are in the units of the number of amino acid substitutions per site.

### Heterologous expression in ND7/23 cells and electrophysiology

The electrophysiological measurements of NaRs were performed using ND7/23 cells, hybrid cell lines derived from neonatal rat dorsal root ganglia neurons fused mouse neuroblastoma [21,22]. We choose the ND7/23 cells because of high reproducibility of experiments. Human codon-adapted NaR genes were synthesized by Gen Script (Piscataway, NJ, USA) and cloned into peGFP vector between HindIII and BamHI sites. All the constructs were verified by DNA sequencing. Adeno-associated viruses construct where cloned with overlap extension PCR into an AAV plasmid backbone. Between FusionRed (FRed) and the opsin, the trafficking sequence from Kir2.1 was inserted (KSRITSEGEYIPLDQIDINVV). On the C-terminal of FRed the endoplasmatic export sequence (FCYENEV) was introduced for better membrane targeting [23].

ND7/23 cells was purchased from DS Pharma Biomedical (Osaka, Japan) and cultured in high-glucose DMEM media (Wako) in a 37 ^o^C, 5% CO_2_ incubator. Transfection of ND7/23 cells was performed by Lipofectamine 2000 (Invitrogen, Carlsbad, CA, USA). Cells were supplemented with 1 μM all-*trans*-retinal (Sigma) after transfection. Expression was confirmed by an inverted microscope TE2000 (Nikon) equipped with a CSU-W1 confocal scanner unit (Yokogawa) and an EMCCD camera Cascade II (Photometrics)

Whole-cell patch clamp recordings on ND7/23 cells were performed with an Axopatch 200B amplifier (Molecular Devices, Sunnyvale, CA, USA). Continuous light was illuminated by OSG L12194-00-39070 (Hamamatsu Photonics, Shizuoka, Japan) via a light guide into an inverted microscope, IMT-2 (Olympus, Tokyo Japan). Illumination was controlled by a mechanical shutter LS6S with an opening time of 0.7 ms and an closing time of 0.8 ms (Vincent Associates, Rochester, NY, USA). Glass pipettes were fabricated by a micropipette puller, P-97 (Sutter Instrument, Novato, CA, USA) and fire-polished by using a micro forge, MF-830 (Narishige, Tokyo, Japan). The pipette resistance was between 1.5–2.5 MΩ. The pipette electrode was controlled by a micro manipulator, PCS-5000 (Burleigh instruments, Fishers, NY, USA). Current traces were recorded at 10 kHz and filtered to 2 kHz by an internal circuit of the amplifier. Data acquisition, shutter triggering were performed by pClamp 10 software via a Digidata 1550 (Molecular Devices, Sunnyvale, CA, USA). Data were analyzed by Clampfit and Origin software.

The standard external solution contained 140 mM NaCl, 2 mM MgCl_2_, 2 mM CaCl_2_, 2 mM KCl, 10 mM Hepes-NaOH (pH 7.2). The standard internal solution contained 110 mM NaCl, 2 mM MgCl_2_, 1 mM CaCl_2_, 5 mM KCl, 10 mM EGTA, 10 mM Hepes-NaOH (pH 7.2). Osmolality of the solutions were adjusted to 300 mOsm by adding appropriate amount of sucrose.

### Heterologous expression in *E*. *coli* and pumping assay

Expression and isolation of NaRs were performed as described previously [13]. In short, *E*. *coli* expressing rhodopsins were harvested by centrifugation (4,800 × g, 3 min), washed for three times and resuspended in aqueous solution containing 100 mM salt (NaCl or KCl). 7.5 mL of cell suspension at OD _660_ = 2 was placed in the dark and then illuminated at *λ* > 500 nm by a 1-kW tungsten–halogen projector lamp (Rikagaku, Japan) through a glass filter (Y-52, AGC Techno Glass, Japan). The light-induced pH changes were measured by a pH electrode (HORIBA, Japan).

### Spectroscopy

The time evolution of the transient absorption change of photo-excited NaRs was observed as previously described [13]. The purified sample was resuspended in buffer containing 50 mM Tris-HCl (pH 8.0), 100 mM NaCl and 0.1% DDM. The sample solution was placed in a quartz cuvette and it was illuminated with a beam of second harmonic of a nanosecond pulsed Nd^3+^-YAG laser (λ = 532 nm, INDI40, Spectra-Physics). The excitation laser power was 3 mJ/(cm^2^_•_pulse). Sample solution of 0.6 mL was used for the measurement. The transient absorption change was obtained by observing the change of the intensity of monochromated output of a Xe arc lamp (L9289-01, Hamamatsu Photonics., Japan) passed through the sample by a photomultiplier tube (R10699, Hamamatsu Photonics, Japan) after photo-excitation. The transient absorption spectra were reconstructed from the time-evolution of transient absorption change at various wavelengths from 360 to 710 nm with 10-nm interval. The signals were global-fitted with a multi-exponential function and decay-associated spectra were obtained by plotting the pre-exponential factor against probed wavelengths [13].

### Hippocampal neuron culture and electrophysiology

#### primary hippocampal neuron culture and viral transduction

Primary cultured hippocampal neurons were prepared from male and female P0 Sprague-Dawley rat pups (Envigo). CA1 and CA3 were isolated, digested with 0.4 mg ml^−1^ papain (Worthington), and plated onto glass coverslips precoated with 1:30 Matrigel (Corning). Cultured neurons were maintained in a 5% CO_2_ humidified incubator with Neurobasal-A medium (Invitrogen) containing 1.25% fetal bovine serum (FBS, Biological Industries), 4% B-27 supplement (Gibco), 2 mM Glutamax (Gibco). To inhibit glial overgrowth, 2 mg ml^−1^ fluorodeoxyuridine (FUDR, Sigma) was added after 4 days of *in vitro* culture (DIV). Three microliters of viral suspension carrying an NaR-ts-FRed-er or eArch-ts-FRed-er (AAV2/1.hSyn1.KR2-ts-FRed-er.WPRE, AAV2/1.hSyn1.SrNaR-ts-FRed-er.WPRE, AAV2/1.hSyn1.FdNaR-ts-FRed-er.WPRE, AAV2/1.hSyn1.NyNaR-ts-FRed-er.WPRE or AAV2/1.hSyn1.Arch-ts-mCherry-er.WPRE) were added at 5 DIV. Cultured neurons were used between 12–17 DIV for experiments.

#### Confocal imaging

Cover slips carrying neuronal cultures were fixed with 4% PFA and mounted onto an object slide with PVA-DABCO mounting media (Sigma). Images were acquired on a Zeiss Confocal 710 with oil immersion 63x objective with 1.4 N.A. Images stacks were captured at 1024 x 1024 with an axial spacing of 6 micrometers Presented images are maximum intensity projections of three consecutive z-planes. All images are shown in 8 bit.

## Results

### Expression screening of NaRs in mammalian cells

**Fig 1:**
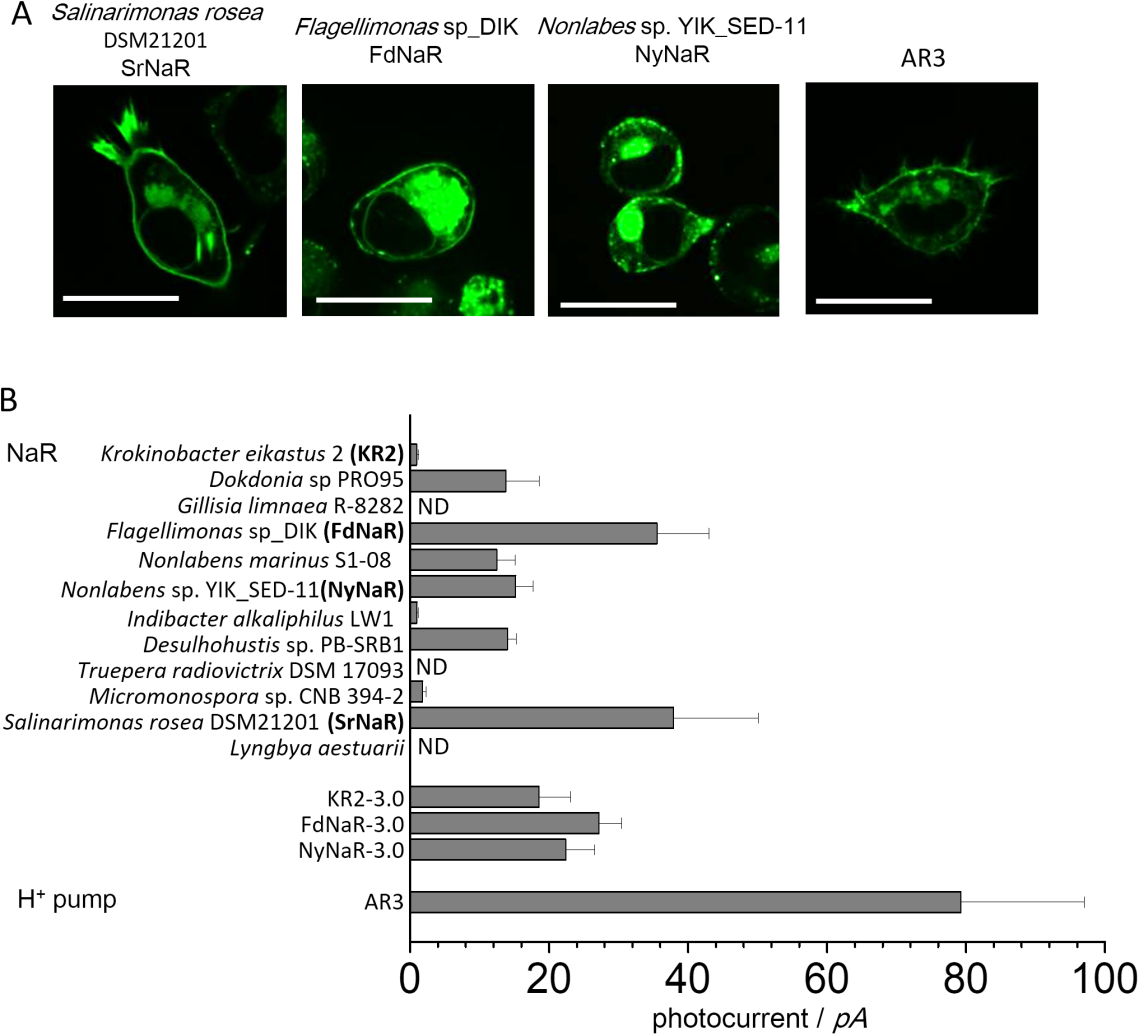
Expression screening of 12 different NaRs in mammalian cultured cells. A, Confocal images of 3 strong expressed NaRs in ND7/23 cells. NaRs were tagged with eGFP at the C-terminus. Scale bar = 25 μm. B, Na^+^ pump currents amplitudes in ND7/23 cells at 0 mV holding potential in a whole-cell patch configuration (N = 4–10). Photocurrents were evoked with 0.8 mW/mm^2^ irradiance at 520 nm except for SrNaR (550 nm) and AR3 (560 nm). Data represent the mean ± SEM (standard error of the mean). ND: Not detected.

Searching genomic database of flavobacteria for opsin-related sequences with a typical NaRs sequence motif revealed 12 candidate genes. The phylogenetic tree is shown in S1 Fig. Amino acid alignment of helix-C region which is given in S2 Fig reveals the characteristic arrangement of the NaRs specific amino acids glutamine (N), aspartate (D) and glutamate (Q). We synthesized 12 genes in full length containing these sequence pattern. The codon usage is optimized for human. All the genes are subcloned into peGFP-N1 vector, one of the standard expression vectors for mammalian cells (Fig A in S3 Fig). After 24–36 hours of transfection into ND7/23 cells, we visualized expression of NaRs as GFP fluorescence as well as phase contrast images to emerge cell shape under a confocal microscope. All the images are exhibited in Fig B in S3 Fig. We found that the membrane localization pattern of GFP signal varies among 12 NaRs tested. As mentioned above, GFP fluorescence by KR2 construct is mainly seen in cytoplasmic region of the cell. But 3 NaRs namely *Flagellimonas* sp_DIK (FdNaR), *Nonlabens* sp._YIK-SED-11 (NyNaR) and *Salinarimonas rosea* DSM21201 (SrNaR), showed higher degree of fluorescence in the membrane even though fluorescence can still be seen in the cytosol [24](Fig 1A).

Next, we performed a whole-cell patch clamp measurement of all 12 NaRs. The photocurrent amplitudes at 0 mV under standard ionic condition (see Materials and Methods) are shown in Fig 1B. At high concentration of NaCl in the pipette (110 mM), the NaRs transport Na^+^ rather than H^+^ [16]. Three NaRs from *Gillisia limnaea* R-8282, *Truepera radiovictrix* DSM 17093 and *Lyngbya aestuarii* displayed no photocurrent under various wavelength of light, consistent with their poor membrane expression (Fig B in S3 Fig). Under this condition KR2 as well as NaRs from *Indibacter alkaliphilus*, and *Micromonospora* sp. CNB 394–2 exhibited small photocurrent in the range of 1 to 2 pA, which is also in agreement with their weak fluorescence signals at the plasma membrane (compare Fig 1B and Fig B in S3 Fig). The other 6 NaRs exhibited larger photocurrents between 15–40 pA at their appropriate wavelength of light, although they were all smaller than photocurrents recorded in cells expressing the H^+^-pumping AR3 (also called eArch3.0). Overall the largest photocurrents recorded were displayed by SrNaRs, in agreement with strong membrane-associated fluorescence in cells expressing this rhodopsin. Based on this initial screen of membrane targeting and photocurrent amplitude, we decided to further investigate the pumping characteristics of FdNaR, NyNaR and SrNaR. It has been reported that KR2 constructs carrying an ER export signal and eYFP exhibited larger photocurrents in cortical neurons and cultured cells [17,25]. We therefore replaced the C-terminally fused GFP coding sequence in our rhodopsin expression constructs with an ER export signal and eYFP (namely KR2-3.0, FdNaR-3.0 and NyNaR-3.0 (Fig A in S3 Fig). (We did not test it with SrNaR because of the slow photocycle described later.) In ND7/23 cells transiently transfected with these constructs, we observed improved eYFP fluorescence at the plasma membrane (Fig B in S3 Fig). Larger photocurrents were observed from cells expressing KR2-3.0 compared to the original construct of KR2-eGFP (Fig 1B). However, for FdNaR and NyNaR, no significant difference in photocurrent amplitude was seen between GFP version and 3.0 version (Fig 1B).

### Characterization of Na^+^ pump function

**Fig 2:**
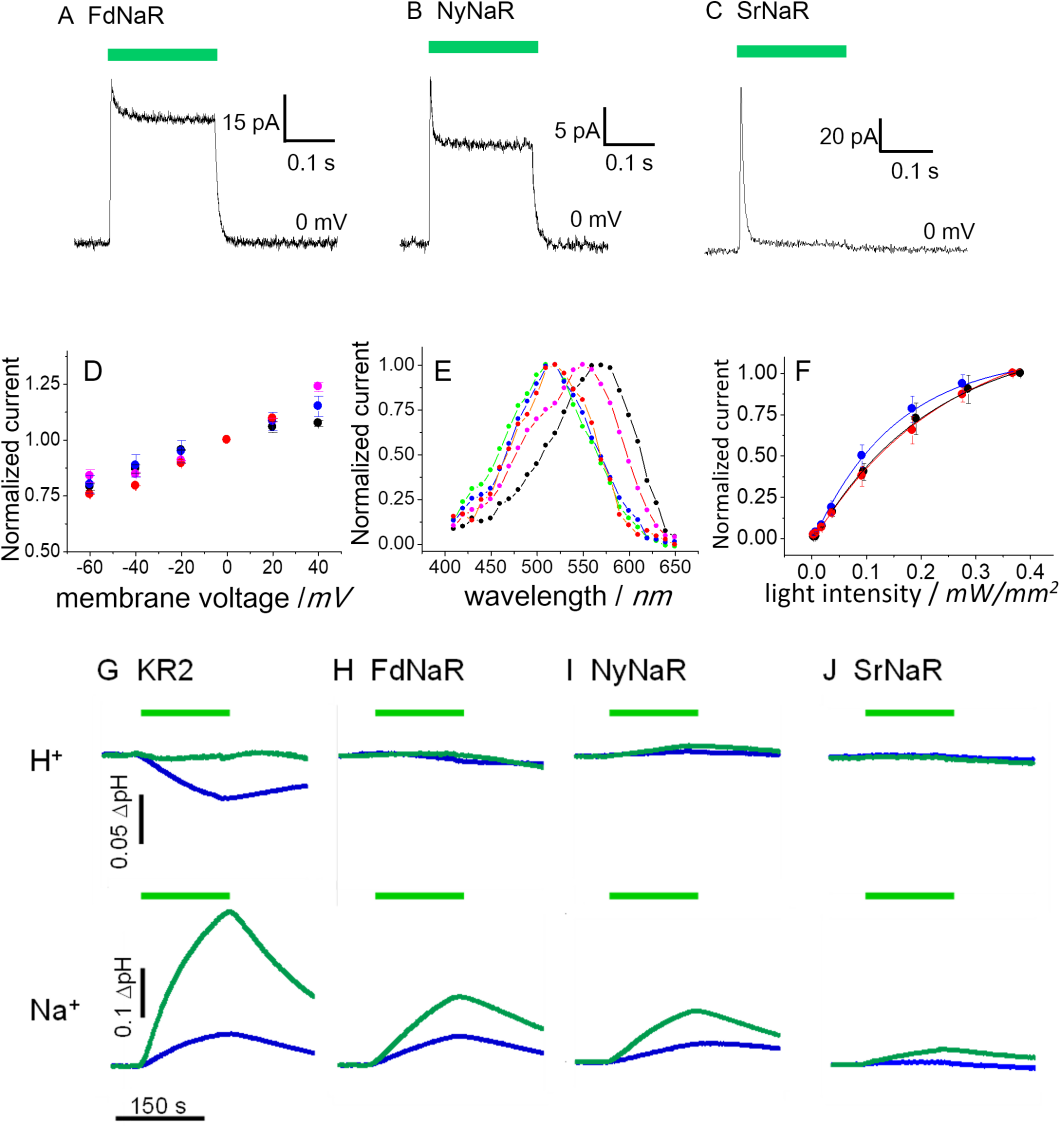
Basic characteristics of pumping functions. (A)-(C), representative photocurrent of 3 NaRs (GFP versions). Membrane voltage was held at 0 mV. 520 nm light (A, B) or 550 nm (C) light (0.8 mW/mm^2^) was illuminated during the time indicated by a green bar. (D) Current-voltage relation (I/V plot) of AR3 (black), FdNaR (blue), NyNaR (red) and SrNaR (purple). (E) Action spectrum, wavelength dependency of photocurrents. green: KR2, blue: FdNaR, red: NyNaR, purple: SrNaR, black: AR3. The light intensities at all wavelengths were 0.2 mW/mm^2^. (F) Light sensitivity of photocurrents from AR3 (black), FdNaR (blue) and NyNaR (red). Data represent the mean ± SE. G-J, H^+^ and Na^+^ transport activity of each NaR measured by a pH electrode after expression in *E*.*coli*. The cells were illuminated (0.14 mW/mm^2^) between 0 and 150 sec. The measurement was performed in the absence (blue) and presence (green) of CCCP.

Fig 2A–2C depicts representative photocurrent records from cells expressing FdNaR, NyNaR and SrNaR (GFP versions). A rectangular light-on stimulation induced a transient current peak which inactivates into different degrees of steady state current for the different NaRs. Upon light-off the photocurrent relaxed to baseline with a time constant of ~ 10 ms. Especially SrNaR reveals an extreme current profile compared with the other two NaRs—a sharp transient peak current that inactivates nearly back to baseline with only residual steady state photocurrent. Fig 2D shows current-voltage relationship (I-V plot) of three NaRs and AR3, a proton pumping rhodopsin. Stationary current of FdNaR and NyNaR and AR3, and peak current of SrNaR were plotted. All the current is linearly increased as the applied voltage rises in the measured condition (-60 to +40 mV). But the slopes are not identical among three NaRs and AR3 suggesting that the different voltage sensitivity on the photocycles. Action spectra, wavelength dependency of photocurrent, are shown in Fig 2E. As expected from UV-vis absorption spectrum [13], KR2 peaked at about 520 nm (green). Also FdNaR and NyNaR displayed maximal photocurrent at approximately 520 nm (blue and red). Photocurrents from SrNaR peaked at around 550 nm (purple) and is therefore ~ 30 nm red-shifted than the other 3 NaRs and 15 nm blue-shifted compared to AR3 (black). Interestingly we observed a shoulder at about 500 nm on the SrNaR spectrum.

Light sensitivity of steady state photocurrent of FdNar, NyNaR and AR3 showed saturation curve at high light intensity (Fig 2F). To evaluate the proton conductance NaRs at different ion compositions, we measured the extracellular medium in an *E*. *coli* suspension expressing the individual NaR with pH sensitive electrode. We first tested H^+^ pumping function in the absence of NaCl (upper trace of Fig 2G). The solution was acidified during illumination, indicating H^+^ export by KR2 from *E*. *coli* cell (blue line), which is diminished when CCCP was present (green line). The new NaRs described here exhibited very low (FdNaR) or almost no H^+^ pumping activity (NyNaR and SrNaR) in this experimental system (upper panels of Fig 2H–2J). We next measured Na^+^ pumping function in the presence of NaCl in solution (Bottom traces of Fig 2G–2J). The pH value was alkalized upon illumination, which is interpreted as a secondary proton uptake after pumping out Na^+^ by NaR from the cells. Such response is further enhanced in the presence of CCCP, because the cells are capable to carry more H^+^ into the cells. The Na^+^ pumping function from this assay indicates that all of 4 NaRs transport Na^+^. The activity is the highest in KR2-expressing cells (Fig 2G bottom). The FdNaR and NyNaR showed moderate activity (Fig 2H and 2I bottom), whereas that of SrNaR was very small (Fig 2J bottom). Previous study showed that the native cells of NyNaR, *Nonlabens* sp. YIK11 exhibit strong light-induced pumping activity both for Na^+^ and H^+^, whereas only proton pumping property was observed in *Krokinobacter eikustus* (KR1 and KR2)[24]. This suggest that expression pattern of pumping rhodopsins vary among the native organisms.

### Spectroscopic properties

**Fig 3:**
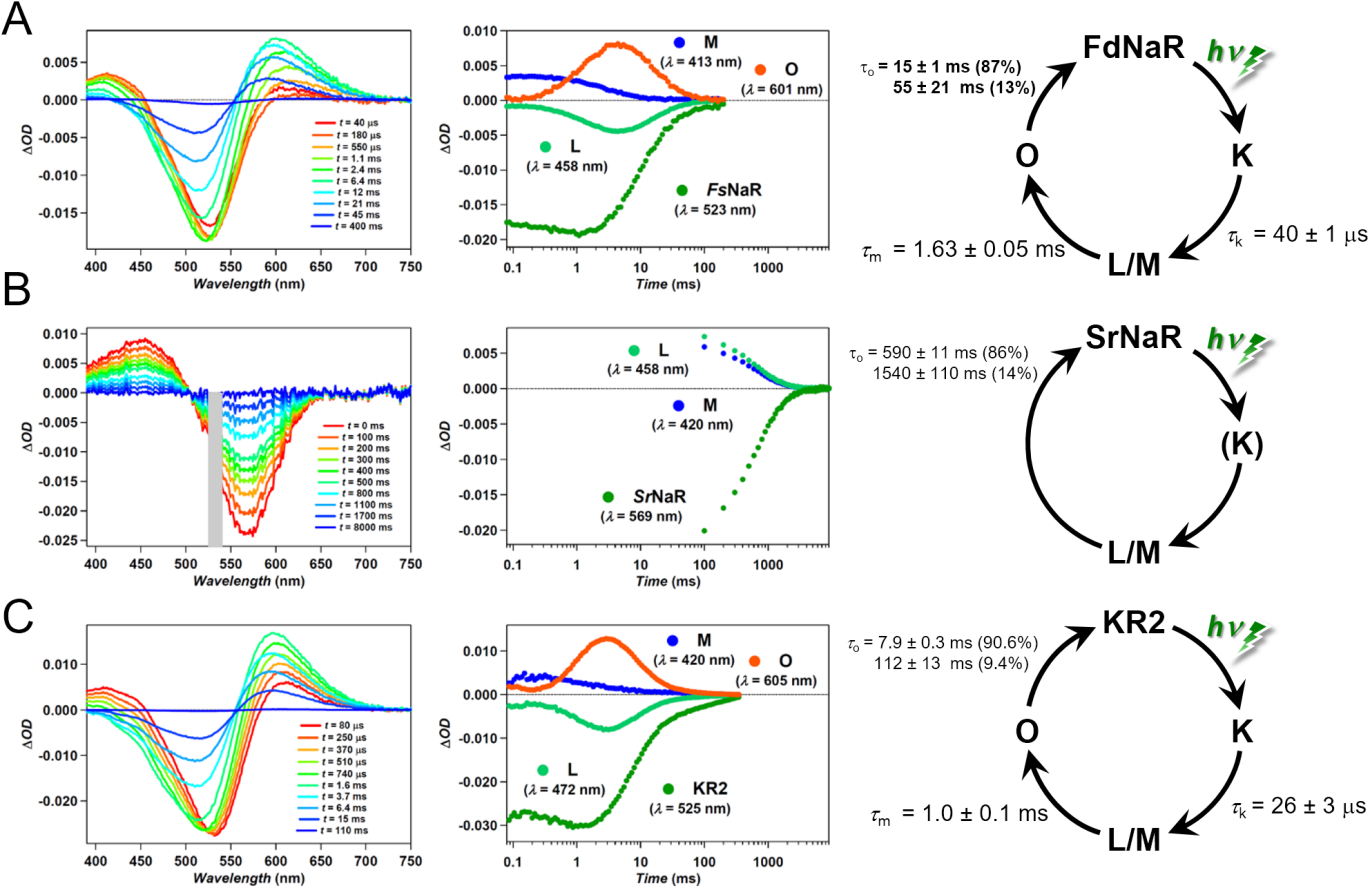
Spectroscopic characteristics of FdNaR, SrNaR and KR2. Transient absorption spectra (left), time evolutions of transient absorption changes (middle) and photocycle schema of FdNaR (**A**), SrNaR (**B**) and KR2 (**C**) reconstituted in DOPC in 100 mM NaCl (pH 8.0). The results of KR2 were reported from [13]. Although the K was not observed in the results for SrNaR, it is expected to exist immediately after absorption of light as FdNaR, KR2 and all other microbial rhodopsins.

From the results above, we confirmed that FdNaR, NyNaR and SrNaR are light-driven Na^+^ pump. In the aspects of photocurrent amplitude and action spectrum, FdNaR and NyNaR are similar to KR2, but rather different is SrNaR. To get an insight into this difference we studied flash photolysis of FdNaR and SrNaR to elucidate their photocycle intermediates and compare them with those of KR2 (Fig 3). FdNaR and SrNaR were purified from *E*.*coli* by the same procedures as KR2 purification [13]. We measured transient absorption spectra in detergent. The lifetime of intermediates of FdNaR was generally similar but slightly slower than those of KR2, i.e. τ_k_ = 26 μs (KR2), 40μs (FdNaR) τ_m_ = 1 ms (KR2), 1.63 ms (FdNaR), and τ_o_ = 7.9 and 112 ms (KR2), 15 and 55 ms (FdNaR). On the other hand SrNaR behaved very different. We only observed a very slow decay of L/M intermediate which mirrors with a recovery of the dark state, being fitted double exponentially (τ = 590 and 1540 ms). No accumulation of O intermediate was detected.

### Application for optogenetics

**Fig 4:**
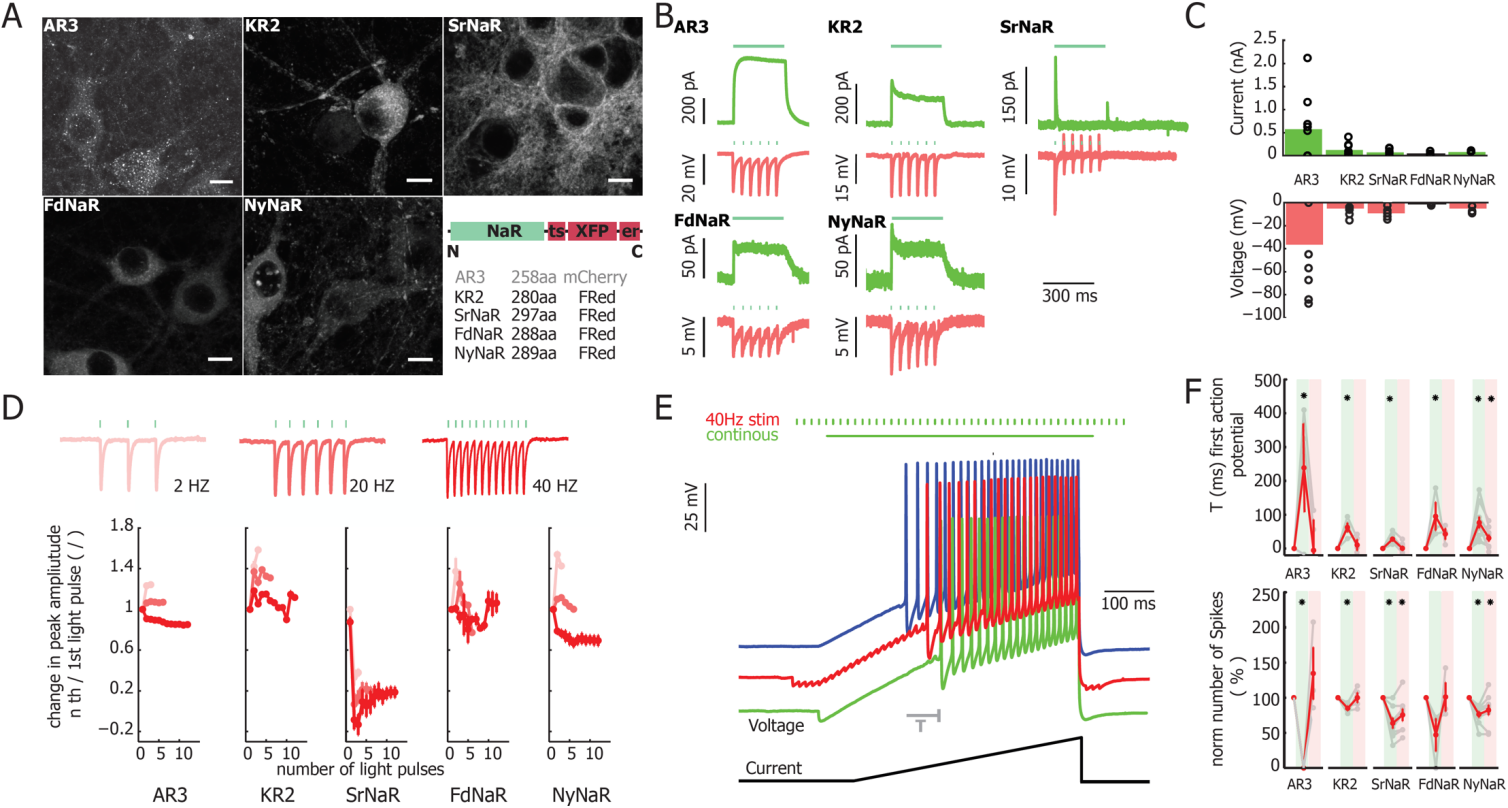
Expression and functional characterization of NaR rhodopsins as optogenetic silencing tools in mammalian neurons. (A) Confocal images of AAV-transduced neurons with different NaR pumps in comparison with the targeting-enhanced proton pump Arch-3.0 The scale bar is 10 μm. Schematic representation of fusion constructs with fluorophore and trafficking sequence (*ts*, and endoplasmatic export sequence (*er*,). (B) Typical photocurrent traces in voltage-clamp shown in *green* under continuous light stimulation (300 ms, 532 nm, 15–20 mW/mm^2^). Corresponding current-clamp recording at 20 Hz light stimulation (3 ms pulse width, 300 ms, 15–20 mW/mm^2^) are shown in *red*. (C) Upper bar diagram depicts population data of average peak photocurrent of NaR compared to AR3. Lower panel represents the amplitude of hyperpolarization relative to resting membrane potential. (D) Adaptation of peak photocurrent upon repetitive light stimulation (3 ms light pulses for 300 ms at 15–20 mW/mm^2^ at 2 Hz, 20 Hz and 40 Hz at different shade of *red*). Example traces are shown in the *upper panel* for FdNaR. Population data for the different constructs are shown in the lower panel (n > 5 cells). (E) Current ramps are used to evaluate optical silencing capability of neuronal activity for NyNaR. During current ramps (2 pA ms^-1^ for 500 ms) either continuous light (*green*), 40 Hz stimulation (*red*) or no light (blue) stimulation was applied. Efficiency of optical silencing was measured both as a shift in time of first action potential (T) and as the change in number of evoked action potentials. (F) Summary of optical silencing efficacy using the two light stimulation protocols (two-tailed Student t-test). All error bars are given as SEM (standard error of mean).

Due to their rapid, Na^+^-specific photocurrents, NaRs could potentially serve as inhibitory optogenetic tools, overcoming the limitations associated with changes in pH [26] or Cl^-^ reversal potential [27] resulting from activation of archaerhodopsins or halorhodopsins, respsectively. To test the activity of NaRs in neuronal context we expressed all three novel NaRs as well as KR2 in a cultured mammalian neurons, and compared their excitability with a commonly used optogenetic inhibitory tool, AR3. To improve membrane trafficking and minimize ER-associated aggregates we fused opsin genes in frame with the Kv2.1 golgi export (ts) and ER export (er2) targeting sequence (Fig 4A)[23]. To minimize opsin activation when observing fluorescence we used a red-shifted fluorophore FusionRed (FRed). As shown in Fig 4A, the fluorescence is most apparent in the soma as well as in neurites while low fluorescence intensities is observed in the nucleus. SrNaR as well NyNaR showed a strong distribution in axonal and dendritic compartments. All NaRs exhibit an outward flux of Na^+^ upon continuous illumination with 532 nm. Hyperpolarization of membrane potential could be induced with a pulsed stimulation paradigm, in example with 20 Hz green light pulses (Fig 4B and Fig 4D). Repetitive stimulation with different frequencies caused a reduction in peak amplitudes as shown in Fig 4D. Photocurrents in neurons expressing SrNaR showed a strong inactivation during repetitive stimulation. To evaluate the efficiency of optical silencing of action potentials, we employed a current ramp protocol combined with two different light delivery paradigms as shown in Fig 4E. In response to continuous illumination, all NaR showed a significant increase in latency to the first action potential. Nevertheless, the efficiency of silencing was 3–5 times less efficient than in neurons expressing eAR3 (Fig 4F). Interestingly, both SrNaR and FdNaR showed a significant reduction of the number of spikes for pulsed light stimulation (Fig 4F, lower panel).

## Discussion

NaR is a new member of the microbial rhodopsin family, actively transporting Na^+^ and H^+^ depending on the ionic conditions. Although spectroscopic and x-ray studies proposed a Na^+^ transport mechanism [17,18], their biophysical properties, including the voltage sensitivity of photocycle intermediates, ion selectivity and the origin of the cation driving force, remain poorly understood. To address these issues, one of the ideal methods is electrophysiology by which ion transport function can be recorded under control of membrane voltage and ionic environment of both sides of cellular membrane with a high time resolution up to ~10 μs. However, the performance of the system greatly relies on the functional expression of a transporter of interest in mammalian cells. Functional membrane-localized expression is also critical for optogenetic application. In the case of channelrhodopsins, use of ChR2, but not ChR1, resulted in successful control of neural excitability because of the ideal expression in neurons [28–30]. Further efforts were made to enhance the performances of ChRs [31,32]. As for H^+^ or Cl^-^ pumping rhodopsins, several screening studies revealed that expression and pumping activities in mammalian cells vary greatly among many microbial rhodopsins from archaea, bacteria, plants and fungi [33,34]. Here we explored 12 NaRs from different sources. The expression patterns and photocurrent amplitudes showed great variation, despite the high sequence homology (20% total identity). Fortunately we were able to identify three candidates (FdNaR, NyNaR and SrNaR) for further investigation. In addition, improvement was made for KR2, when the fluorescence tag and a signaling motif was replaced in the original construct [25]. But this was not the case with FdNaR and NyNaR (Fig 1B). Protein expression levels and the degree of membrane localization therefore depend not only on the protein itself, but also on the fused moiety.

Distinct characteristics of SrNaR observed in the patch-clamp record is the transient photocurrent (Fig 2C). Such current shape was observed in bacteriorhodopsin mutant D96N in which the photocycle is slowed down by two orders of magnitude because of lack of H^+^ donor [35]. Thus we anticipated that the photocycle of SrNaR is much slower than those of FdNaR, NyNaR and KR2. This was indeed proven by the flash photolysis measurement (Fig 3). It is enigmatic that SrNaR as a wild type has such a poor function, pumping only 4 Na^+^ per min (i.e. one photocycle takes about 15 sec). This value would be too small to establish an electrochemical gradient in the native cell. Based on our findings, we speculate that, (1) SrNaR acts as a sensory photoreceptor like SRI and SRII which trigger signaling cascade for phototaxis reaction in the organisms and the Na^+^ pumping is thus only residual property in evolution. It was reported that SRII retains small H^+^ pumping activity [36]. Or (2) The photocycle could be accelerated in its endogenous environment, i.e. under high salt condition (salt rock) where the *Sarinalimonas rosea* was discovered.

The linear relationship between the applied voltage and the current amplitude indicates that the photocycles of FdNaR, NyNaR and SrNaR involve dΨ-dependent intermediates (Fig 2S)[9]. However it was reported that the I/V relation of KR2 showed no apparent voltage dependency between -70 and +50 mV [17]. This difference might derive from a binding affinity at Na^+^ binding site(s), being responsible for either uptake or release of Na^+^ from/to the bulk solution. It was reported that the slope of the I/V plot is influenced by extracellular pH or amino acid replacement in H^+^ transport pathway in H^+^-pumping rhodopsins (GR and CsR) [12,37]. Further assessment of I/V relation should be performed in different ionic conditions in wider voltage range to understand the effect on the photocycle of NaRs.

The pumping assay using *E*. *coli* cell with a pH electrode enables us to make a rough estimation of selectivity between Na^+^ and H^+^. Surprisingly the H^+^ pumping activity is very weak or almost zero in FdNaR and NyNaR, while retaining reasonable Na^+^ pumping signals. This means that these two NaRs might be more Na^+^ selective than KR2 in which the transport ratio of H^+^/Na^+^ is greater than 8000 [16]. The high Na^+^ selectivity of FdNaR and NyNaR could be advantageous for distinct optogenetics application. Despite the stronger silencing capabilities of enhanced proton pumping AR3, the NaRs have the potential to silence neuronal activity in a more physiological manner without changing intra and extracellular pH [26,27,38].

In summary, we compared functionality and photochemical properties of NaRs from different sources. Similarities and differences in aspects of performance in mammalian cells, ion selectivity, photocycle rate and λ_max_ are elucidated. It remains to be studied how these characteristics are achieved. As mentioned above, detailed electrophysiological measures with various ionic environments are necessary for better understanding the vectorial transport. Such studies would provide significant information whether dpH and dpNa contribute equally to the photocycle. With the help of X-ray structure [17,18], mutation studies would also help understand mechanism of the light-driven Na^+^ pump. The knowledge will allow us to design new optogenetics tools such as color variants, cation variants, and a light-gated Na^+^ selective channel.

## Supporting information

S1 Fig Evolutionary relationships of NaR. A phylogenetic tree of 12 NaRs tested in this study are depicted. Bacteriorhodopsin from *Halobacterium Salinarium* (BR) is also included. Accession numbers in Gene bank are, *Krokinobacter eikastus* (KR2): AB738960.1, *Dokdonia* sp. PRO95: JN827400.1, *Gillisia limnaea R-8282*: EHQ02967.1, *Flagellimonas* sp_DIK: KM461123.1, *Nonlabens marinus S1-08*: KJ019877.2, *Nonlabens* sp.YIK-SED-11: KJ019875.2, *Indibacter alkaliphilus LW1*: EOZ93469.1, *Desulhohustis* sp. PB-SRB1: ESQ10031.1, *Truepera radiovictrix DSM17093*: ADI16038.1, *Micromonospora* sp. CNB 394–2: WP_018784639.1, *Salinarimonas rosea DSM21201*: WP_052341415.1, *Lyngbya aestuarii*: WP_052001698.1, *Halobacterium salinarum* (BR): AAA72504.1
(TIF)

S2 Fig A key motif for ion selectivity in the helix C. Amino acid sequence alignment of the helix C region of 12 NaRs are shown with those of bacteriorhodopsin and halorhodopsin. Three characteristic amino acids (N, D and Q) in NaRs are indicated by red arrowheads.
(TIF)

S3 Fig Expression pattern of NaRs in ND7/23 cells. (A) NaR construct with eGFP or ts-eYFP-er. (B) Microscopic images of NaRs. Fluorescent images are acquired by confocal mode (left) and cell shapes are observed by phase contrast mode (right). 60x objective, scale bar = 25 μm.
(TIF)
