# Supplementary figures and images for: Functional characterization of sodium-pumping rhodopsins with different pumping properties
Source: PLoS One. 2017 Jul 27;12(7):e0179232. doi: 10.1371/journal.pone.0179232 (PMC5531490; doi:10.1371/journal.pone.0179232)

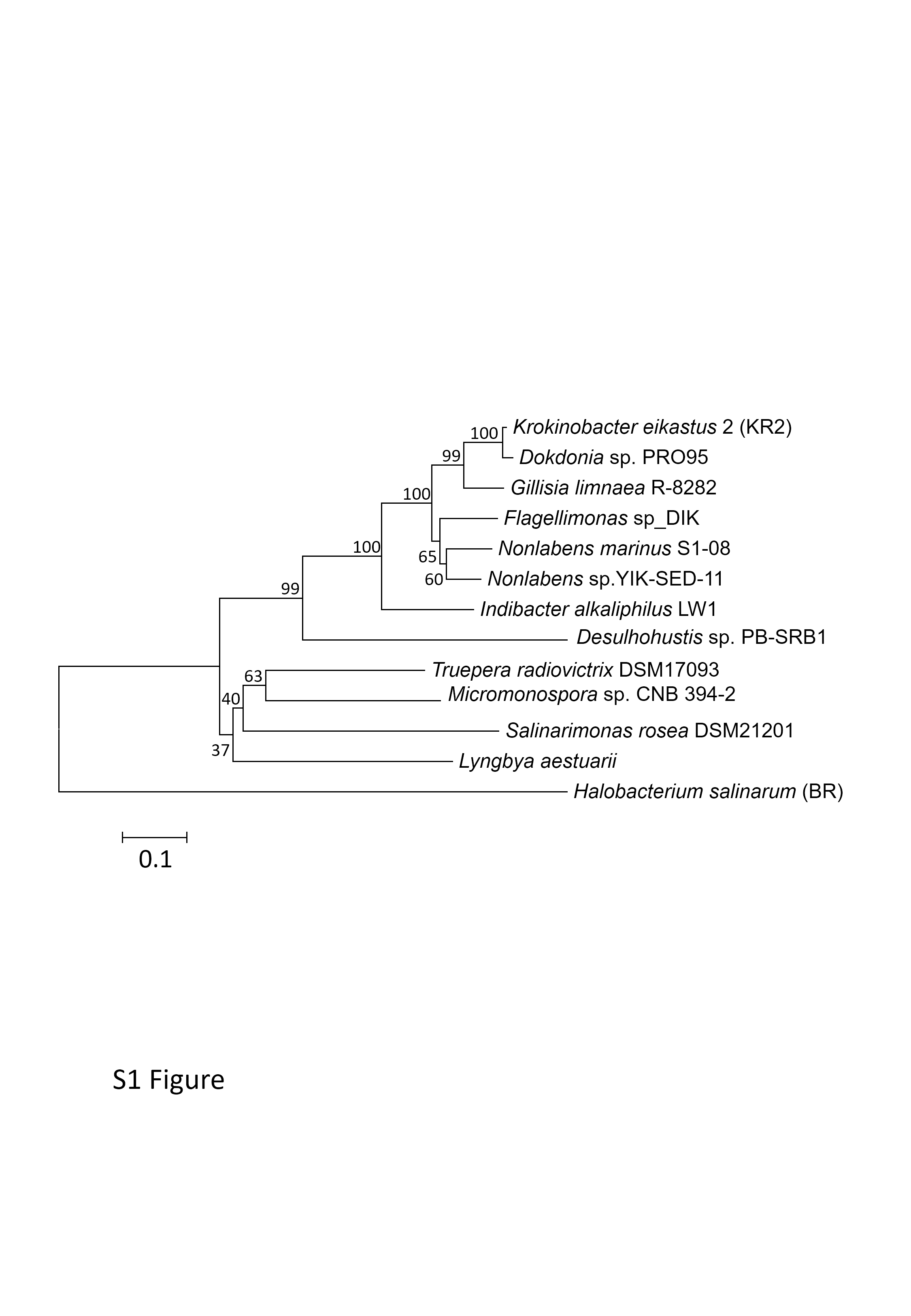

Supplement: S1 Fig — A phylogenetic tree of 12 NaRs tested in this study are depicted. Bacteriorhodopsin from Halobacterium Salinarium (BR) is also included. Accession numbers in Gene bank are, Krokinobacter eikastus (KR2): AB738960.1, Dokdonia sp. PRO95: JN827400.1, Gillisia limnaea R-8282: EHQ02967.1, Flagellimonas sp_DIK: KM461123.1, Nonlabens marinus S1-08: KJ019877.2, Nonlabens sp.YIK-SED-11: KJ019875.2, Indibacter alkaliphilus LW1: EOZ93469.1, Desulhohustis sp. PB-SRB1: ESQ10031.1, Truepera radiovictrix DSM17093: ADI16038.1, Micromonospora sp. CNB 394–2: WP_018784639.1, Salinarimonas rosea DSM21201: WP_052341415.1, Lyngbya aestuarii: WP_052001698.1, Halobacterium salinarum (BR): AAA72504.1 (TIF) [file pone.0179232.s001.tif]

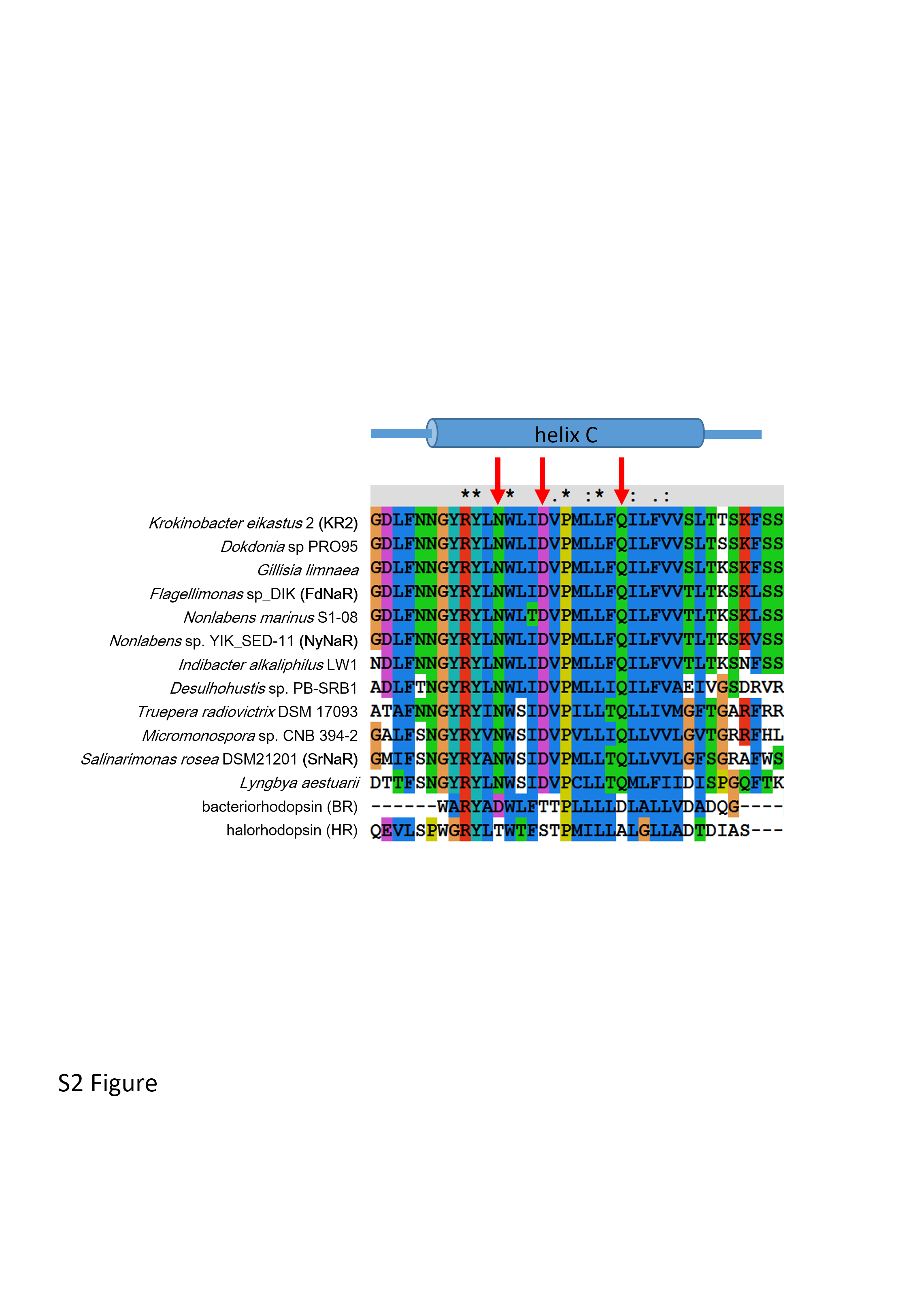

Supplement: S2 Fig — Amino acid sequence alignment of the helix C region of 12 NaRs are shown with those of bacteriorhodopsin and halorhodopsin. Three characteristic amino acids (N, D and Q) in NaRs are indicated by red arrowheads. (TIF) [file pone.0179232.s002.tif]

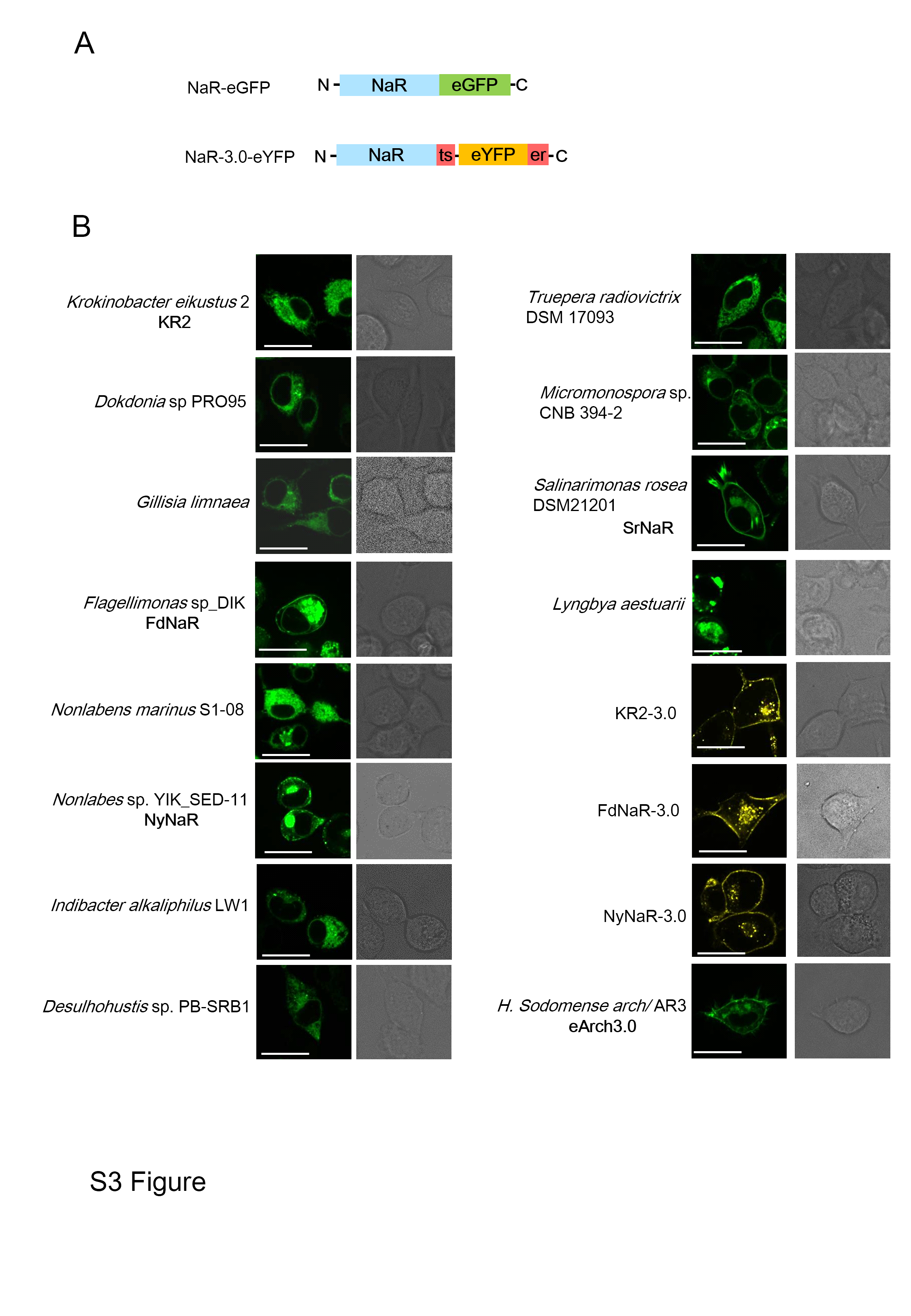

Supplement: S3 Fig — (A) NaR construct with eGFP or ts-eYFP-er. (B) Microscopic images of NaRs. Fluorescent images are acquired by confocal mode (left) and cell shapes are observed by phase contrast mode (right). 60x objective, scale bar = 25 μm. (TIF) [file pone.0179232.s003.tif]
